# Associations of Suicide Rates With Socioeconomic Status and Social Isolation: Findings From Longitudinal Register and Census Data

**DOI:** 10.3389/fpsyt.2019.00898

**Published:** 2020-01-14

**Authors:** Anatol-Fiete Näher, Christine Rummel-Kluge, Ulrich Hegerl

**Affiliations:** ^1^Department of Pneumology, Helios Klinikum Emil von Behring, Berlin, Germany; ^2^Department of Psychiatry and Psychotherapy, University of Leipzig, Leipzig, Germany; ^3^Department of Psychiatry, Psychosomatics and Psychotherapy, Goethe Universität Frankfurt am Main, Frankfurt am Main, Germany

**Keywords:** suicide, public mental health, social determinants, prevention, socioeconomic status, social isolation

## Abstract

Suicide represents a major challenge to public mental health. In order to provide empirical evidence for prevention strategies, we hypothesized current levels of low socioeconomic status (SES) and high social isolation (SI) to be linked to increased suicide rates in N = 390 administrative districts since SES and SI are associated with mental illness. Effects of SES on suicide rates were further expected to be especially pronounced in districts with individuals showing high SI levels as SI reduces the reception of social support and moderates the impact of low SES on poor mental health. We linked German Microcensus data to register data on all 149,033 German suicides between 1997 and 2010 and estimated Prentice and Sheppard’s model for aggregate data to test the hypotheses, accounting for spatial effect correlations. The findings reveal increases in district suicide rates by 1.20% (p < 0.035) for 1% increases of district unemployment, suicide rate decreases of −0.39% (p < 0.028) for 1% increases in incomes, increases of 1.65% (p < 0.033) in suicides for 1% increases in one-person-households and increases in suicide rates of 0.54% (p < 0.036) for 1% decreases in single persons’ incomes as well as suicide rate increases of 3.52% (p < 0.000) for 1% increases in CASMIN scores of individuals who moved throughout the year preceding suicide. The results represent appropriate starting points for the development of suicide prevention strategies. For the definition of more precise measures, future work should focus on the causal mechanisms resulting in suicidality incorporating individual level data.

## Introduction

Suicide constitutes a substantial public health problem: In Europe, the local age-adjusted numbers amounted to 12.84 deaths per 100,000 individuals in 2016, according to WHO data (1). This constitutes the second highest suicide rate across all WHO global regions. In order to improve the situation, suicide prevention has been given highest priority on the agenda of the European Comission (2). In addition to this, the reduction of suicide mortality has been defined as one of the target 3.4 indicators of the United Nations’ sustainable development goals for 2030 (3).

With regard to prevention, it is of particular significance that sociodemographic characteristics mediate suicide risks: A systematic review of population level studies revealed associations of poverty and suicide rates (4). According to recent studies (5, 6) increases in suicide rates are linked to rising unemployment rates. Moreover, there exists evidence for an inverse relationship between social cohesion and suicide (7–9). Adding to these findings, the aim of this study is to provide empirical evidence on potential associations of individual level indicators of socioeconomic status (SES) and social isolation (SI) with district suicide rates. While suicide itself is a complex phenomenon determined by genetic, cultural and behavioral factors (10, 11), such associations help to identify populations at risk.

Concerning suicide risks linked to SES, a meta-analysis by Li et al. (12) found the highest relative and population attributable risks of committing suicide for males in low ranked occupational classes and for persons with low educational achievement. Among women, relative risk ratios and population attributable risks were highest for unemployed individuals and individuals with low education. Regarding relations of education and suicide, suicides were also 2.12 times more often observed in the lowest educational group as compared to individuals with the highest educational levels across 35 countries in a study combining census and mortality register data (13). Unemployment turned out to be associated with suicide in a more recent meta-analysis (14). Relative risks of these associations were reduced after controlling for prior mental health. Financial strain, as captured by a family income to poverty threshold ratio has been found to be positively related to suicide attempts and ideations in a household survey among U.S. adults (15). As further crucial measures of SES, income-based indicators of individual level inequalities were positively related to suicide risks among both genders in a longitudinal study incorporating official Swedish mortality data (16).

In contrast to these results, several studies yielded less conclusive findings. While the most recent meta-analysis revealed positive associations of suicide with low education and unemployment, the authors regard these as not clinically significant due to small effect sizes (17). Overall, the study concluded that the results warrant further research on the effects of demographics on suicidal behavior. Among Malaysian in-patients, low social class predicted transitions from suicide ideations to attempt whereas unemployment and low educational levels did not confer any significant increases in suicide attempts (18). In addition, contradictory results were reported by Lukaschek et al. (19) who failed to find a significant effect of unemployment on suicides among in-patients in six German psychiatric hospitals. Regarding in-patient samples, it is important to note that these are not necessarily representative of all individuals that die by suicide. The findings by Chan et al. (18) and Lukaschek et al. (19) might possibly not apply to suicide in general.

Low SES does not increase suicide risks per se. On the one hand, there exist links of SES and psychopathology (20–23). On the other, quantitative meta-studies reveal associations of mental disorders and suicide (24, 25), avoiding the methodological problems of psychological autopsy (26). These findings suggest mental-ill health as a possible mechanism that links low SES levels to increases in suicide risks, allowing for causality in two directions. While poor mental health may be triggered by low SES, low SES may also represent the consequence of psychopathology. Several longitudinal studies investigating both explanatory approaches suggest an interaction of mental health and SES over the life course of individuals (27, 28).

Exploring potential mechanisms that explain SES effects on suicide does not lie within the scope of this study. In order to inform public health policies, we tested the hypothesis that lower levels of contemporary SES are correlated with higher suicide rates (Hypothesis I).

Our second hypothesis relates suicides to social isolation (SI). We define SI as a lack of social network quality. In this context, social network quality itself is understood as the extent to which existing ties provide the resources an organism can draw on in order to achieve its life goals (29). SI possibly acts as a root cause for feelings of thwarted belongingness. In combination with perceived burdensomeness, thwarted belongingness is seen as critical to the development of suicide ideation in Joiner’s (30) psychological model of suicide. This, in turn, leads to increased suicide risks in individuals. In accordance with the theory, the empirical evidence on links of SI with increased suicide risks is well established. Proxying for SI in a study incorporating 21,169 suicides, a Danish study revealed associations of single marital status and suicide (31). These results have been confirmed in Sweden (32), the U.S. (33), Great Britain (34), Finland (35), Austria, Belgium, Denmark, Norway and Switzerland (36) and Canada (37). Likewise, Agerbo et al. (38) found a relation between increased risks of suicide and living alone as well as being divorced. Utilizing relocations as SI proxies, several studies found these to be associated with completed and attempted suicides in adolescents (39, 40) and elderly people (41, 42).

There exists a solid body of evidence on associations between SI and mental health outcomes (43–45). Again, this suggests psychopathology as a potential pathway through which SI affects suicidality. It is important to note, however, that individuals suffering from mental illness exhibit impairments in social functioning, which leads to a disruption of personal networks (46). For this reason, not only does SI possibly affect mental health but mental health also impacts on SI levels. As is the case with SES and mental health, associations between SI and mental health outcomes do not determine the direction of causality. While this needs to be addressed with additional studies allowing for causal conclusions, we tested the second hypothesis that higher levels of SI are associated with higher suicide rates (Hypothesis II).

Social support represents a substantial social network resource for individuals in order to cope with stressful life events (47, 48) and has been shown to moderate the impact of environmental conditions on symptoms of poor mental health (49–52). Effects of low SES should thus be aggravated in socially isolated individuals. Hence, we also evaluated if interactions between lower levels of SES and higher levels of SI are linked with higher suicide rates (Hypothesis III).

## Data and Methods

### Data

For the observation period 1997 to 2010, data on suicides were obtained from the German death record edited by the federal statistical office (DESTATIS). The dataset lists all yearly German deaths by cause as coded by the ICD system (53, 54). With regard to these data, it is relevant that German data protection regulations do not permit individual level analyses. Hence, suicide deaths were examined as aggregated rates at the level of administrative districts. It needs to be considered in this respect that, after German reunification, extensive administrative reforms including new territorial definitions of district populations were implemented in Eastern Germany. Reformed areas were therefore grouped to greater regions such that they resembled the territorial status of the observation period’s last year, yielding a total of *N* = 390 districts. Non-reform-associated differences in district specific suicide rates are hereby properly identified.

In order to gain information on the socioeconomic conditions of district populations, the numerical identifiers of districts included in the record data were utilized to merge this dataset with the data provided by the German Microcensus. Based on a random household sample, the Microcensus is the official survey on living conditions in Germany. Selected respondents are legally bound to answer items on education, labor force participation, income, family and housing situation. As it can be inferred from **Table 1**, the survey’s population sample size is large enough to permit district level analyses: In all observation years but 2005, it corresponds to at least 0.74% of the population sizes in 95% of the districts. A drawback of the Microcensus is, however, that one quarter of the observation units is replaced every year. Periods longer than four years are thus not covered by panel data. In order to examine suicide deaths over a longer time frame, the employed dataset was constructed from annually repeated cross sections over the course of *T* = 14 years, i.e. *N* × *T* = 5,460 district observations.

**Table 1:** Summary of microcensus sample sizes per district by year.

Year	Mean	Min.	Max.	5^th^ Perc. of % Dis. Pop.
1997	4,694	305	31,233	0.78
1998	4,640	315	30,381	0.77
1999	4,568	325	29,720	0.76
2000	4,480	322	29,034	0.74
2001	4,473	343	28,732	0.74
2002	4,420	347	28,604	0.75
2003	4,429	328	28,580	0.75
2004	4,403	321	28,491	0.74
2005	4,295	292	27,795	0.59
2006	4,337	332	28,130	0.74
2007	4,207	318	27,034	0.74
2008	4,243	323	27,298	0.75
2009	4,285	320	27,675	0.77
2010	4,256	297	27,369	0.76

Min., Minimum; Max., Maximum; 5^th^ Perc. of % Dis. Pop.: 5^th^ Percentile of Percentage of District Population.

Source: German Microcensus & Area Population Numbers of Germany, 1997 to 2010 - ed.by the German Federal Statistical Office (DESTATIS), own calculations were performed with STATA 15.0.

### Measures and Descriptive Statistics

We define suicide as coded by the ICD 9 categories E950–E959 for 1997 and as the lethal consequence of intentional self-harm corresponding to the ICD 10 codes X60–X84 for the years from 1998 onwards, respectively (53, 54). All 149,033 cases of suicide death according to this definition were extracted from the death record to calculate district specific suicide rates. The different coding categories introduced with the 10*_th_* ICD revision in 1998 may be associated with structural breaks in reported suicide numbers and hence lead to biased estimates of suicide rates. To detect any of these breaks, yearly changes in suicide numbers per district were regressed on a dummy variable indicating the year of the ICD change controlling for district specific time trends and time fixed effects. As no significant results were found ICD 9 and ICD 10 codings of suicide are regarded as equivalent measures.

**Figure 1:**
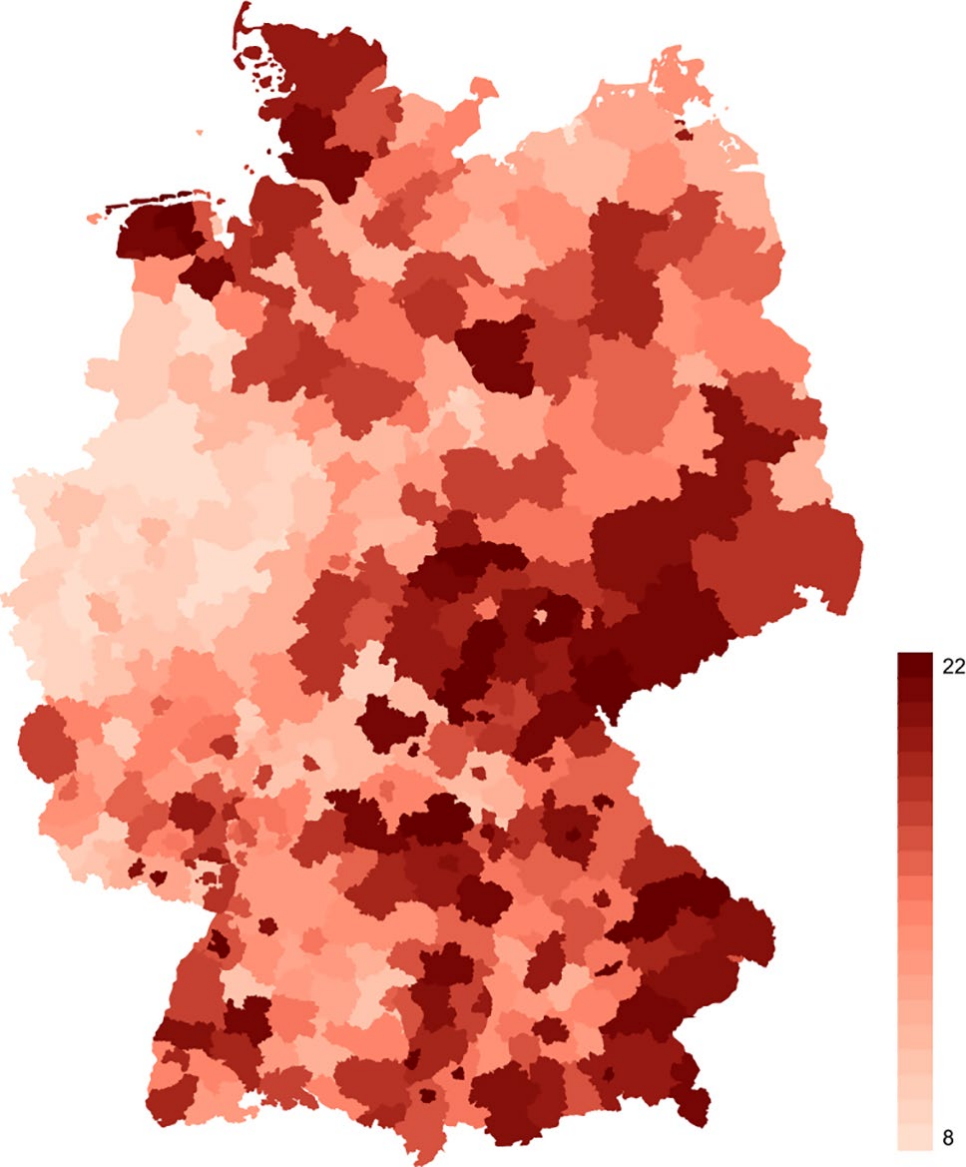
Observation period means of age- and gender-adjusted district suicide rates in Germany. The legend on the right hand side provides color codes for suicide rate values ranging from 8 (minimum) to 22 (maximum) per 100,000 inhabitants. *Source*: German Death Record, Area Population Numbers of Germany, 1997 to 2010, and 2013 European Standard Population - ed. by the German Federal Statistical Office (DESTATIS). Stata 15.0 was used to perform own calculations and create this figure.

Because population structures vary over time and region, suicide rates were age- and gender-adjusted based on the 2013 European standard population to ensure the comparability across all districts. The gender specific distribution of district suicide rates over time can be inferred from **Table 2**. An illustration of the spatial distribution of the observation period means of the adjusted district suicide rates is provided by **Figure 1**.

**Table 2:** Summary of age and gender standardized district suicide rates by year and gender.

Year	Females				Males			
	Mean	S.D.	Min.	Max.	Mean	S.D.	Min.	Max.
1997	4.04	1.90	0.44	12.19	12.95	4.97	2.88	31.07
1998	3.65	1.75	0.54	11.95	12.49	4.42	3.07	31.52
1999	3.69	1.97	0.54	21.46	11.72	4.35	1.66	32.18
2000	3.61	1.80	0.29	11.63	11.69	4.20	2.50	28.06
2001	3.49	1.75	0.55	11.53	11.73	4.42	3.06	26.04
2002	3.47	1.70	0.56	10.87	11.50	4.48	2.87	37.37
2003	3.41	1.66	0.29	13.07	11.50	4.21	1.82	30.71
2004	3.25	1.91	0.00	17.61	10.71	3.77	0.00	28.58
2005	3.25	1.79	0.00	11.71	10.36	4.22	0.00	28.59
2006	2.99	1.71	0.30	11.25	10.04	3.83	2.44	33.44
2007	2.74	1.41	0.35	8.92	9.46	3.71	2.35	25.13
2008	2.97	1.70	0.36	11.96	9.54	3.93	1.43	34.26
2009	2.81	1.67	0.35	14.07	9.67	3.60	1.31	27.48
2010	3.00	1.70	0.42	13.13	9.95	3.56	2.74	29.47

Min., Minimum; Max., Maximum; S.D., Standard Deviation.

Source: German Death Record, 1997 to 2010 - ed. by the German Federal Statistical Office (DESTATIS), own calculations were performed with STATA 15.0.

**Figure 2:**
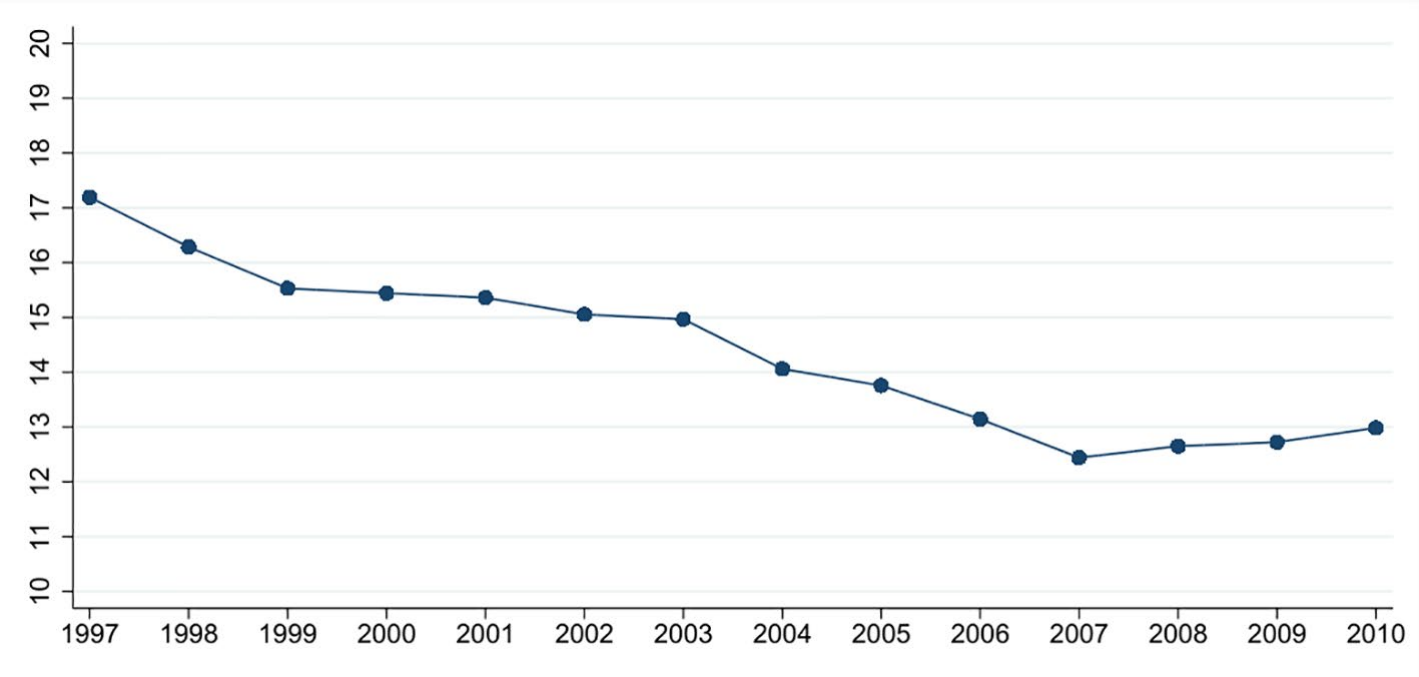
Yearly means of age- and gender-adjusted district suicide rates in Germany. Suicides per 100,000 inhabitants shown on the y-axis, observation years shown on the x-axis. *Source*: German Death Record, 1997 to 2010 - ed. by the German Federal Statistical Office (DESTATIS), STATA 15.0 was used to create this figure.

Furthermore, as can be seen from **Figure 2**, the yearly means of the adjusted district suicide rates in Germany declined between the years from 1997 to 2010. Potential reasons for this consist in better help seeking behaviors and treatments of patients suffering from mental illness, better training of physicians to recognize suicidal behavior and the set up of intervention programs at community levels (55). In order to permit the identification of changes in suicide rates associated with SES and SI, this general linear downward trend in the data was accounted for by including a variable indicating the observation year in the models.

SES was operationalized employing measures for educational achievements, occupational status and log income. Educational achievements were quantified according to the scheme provided by the “Comparative Analyses of Social Mobility in Industrial Nations (CASMIN)” (56). The CASMIN scheme constitutes an internationally comparable measure that distinguishes hierarchical educational classes constructed from combinations of school and vocational degrees. The classes reach from 1*a* (inadequately completed general education) to 3*b* (higher tertiary education) and represent the boundaries of the respective country’s educational system on the one hand and differentiate between required educational levels for certain labor market positions on the other (57).

The “International Socio-Economic-Index of Occupational Status (ISEI)” (58) was applied for the measurement of occupational status. This index relies on a continuous scoring system with values ranging from 16 (low Status) to 85 (high status). For unemployed individuals, scores of the last occupation were utilized. Beyond that, unemployment and, as it is not included in the ISEI scheme, minor employment were recorded in own variables and included in the analysis.

As another significant component of SES, we calculated the log of the equivalent household income from the income variables provided by the Microcensus. The highest psychosocial distress should result from the lowest SES conditions. In order to proxy especially for these situations, two variables indicating the receipt of social benefits due to unemployment (“ALG I/II” in Germany) and the number of received public transfers were added to the models, too.

Similar to previous studies, SI was proxied with two variables on single marital status and living in a one-person-household and a dummy variable recording a possible relocation throughout the year before the survey was conducted (“Moved last Year”). Being divorced or widowed was considered as having a single status in this context.

Age categories as provided with the European standard population were employed for the standardization of suicide rates. In order to control for exponentially increasing suicide risks with age in Germany (59) and continuous age effects, we therefore added continuous measures of age and age squared from the register dataset as controls. **Table 3** summarizes all independent variables over time.

**Table 3:** Independent variables.

Variable	Mean	S.D.	N
ISEI	42.60	15.73	4,598,937
CASMIN	3.40	2.16	7,873,911
No. Public Transfers	0.24	0.51	9,923,291
Age	53.93	4.07	7,324,663
	**Median**	**I.Q.R**.	**N**
Income (household equiv.)	1230.77	1043.71	9,239,242
	**%**	**S.D. (in %)**	**N**
Minor employment	4.00	19.50	7,646,280
Unemployment	4.96	21.71	9,941,490
ALG I/II	4.64	21.03	9,941,494
Moved last year	6.92	25.39	6,438,498
Single	51.88	49.96	9,941,494
One-person-household	17.54	38.03	9,836,123
Gender (male)	73.44	8.78	7,324,663

S.D., Standard Deviation; I.Q.R., Interquartile Range

Source: German Microcensus & Death Record, 12997 to 2010 – ed. by the German Federal Statistical Office (DESTATIS), own calculations were performed with STATA 15.0.

Finally, values of the ISEI, CASMIN and log income variables were inverted and mean-centered for the calculation of the proposed *SES* × *SI* interactions.

### Methods

Combining individual and district level data for the estimation of SES and SI effects on suicide rates represents a source of ecological bias. The problem arises in aggregate data analysis if summary measures are employed to calculate effects on marginal outcomes. Distributions of summaries do not determine individual distributions. Because joint individual distributions have to be specified in order to identify effect parameters, however, estimates depending solely on aggregate data are potentially biased. A first related statistical problem is that, without further assumptions, applying summary measures does not identify the functional form of effects on the individual level. To see this, let:

(1) $$E(y_{idt})=f(\pi_{d}+\beta X_{idt}),$$

where *f* (·) denotes a link function. *y_idt_* is a binary indicator variable, with *y_idt_* = 1 if individual *i* = 1,2,…,*n_dt_* commits suicide in district *d* = 1,2,…,*N* at year *t* = 1,2,…,*T* and *y_idt_* = 0 otherwise. *X_idt_* is a row vector including the controls, the variables on SES and SI and, in order to test Hypothesis III, *SES* x *SI* interaction terms. *β* is a column vector with the respective parameters of interest. Further, *π_d_* represents the district baseline probability of suicides, for *X_idt_* = 0. Given that data on suicides is only available as district specific rates, ${\bar{y}}_{dt}=n_{dt}^{-1}\cdot \sum_{i=1}^{n_{dt}}y_{idt}$ , effects of SES and SI can be estimated by the ecological model

(2) $$E({\bar{y}}_{dt})=f(\pi_{d}+\beta {\bar{X}}_{dt})$$

with ${\bar{X}}_{dt}=n_{dt}^{-1}\cdot \sum_{i=1}^{n_{dt}}X_{idt}$. As it can be seen from equations (1) and (2) the functional form of individual level effects is only correctly specified if *f* (·) is assumed to be linear and completely additive in all of its arguments. It follows in any other case that $n_{dt}^{-1}\cdot \sum_{i=1}^{n_{dt}}f(\pi_{d}+\beta X_{idt})\neq f\left(\pi_{d}+\beta {\bar{X}}_{dt}\right)$. With regard to this, we therefore apply the aggregate data method firstly introduced by Prentice and Sheppard (60). Accordingly, the following equation is being estimated, normalizing the district specific standardized suicide rate *r_dt_* by taking its log:

(3) $$\begin{array}{l} ln(r_{dt})=n_{dt}^{-1}\cdot \overset{n_{dt}}{\underset{i=1}{\sum }}f(\pi_{d}+\beta X_{idt})+\nu_{t}+\kappa_{dt},\\ \kappa_{dt}=\rho W\tau_{dt}+\varepsilon_{dt}, \end{array}$$

with an identity link *f* (·) and a variance fraction *ρ*. The spatial contiguity matrix *W* accounts for a spatial correlation of error components τ*_dt_* among neighboring districts due to omitted district level variables. Spatial correlation effects may result in biased estimates of suicide rates, e.g. if individuals commute between districts that differ in one or more of such unobserved traits. Further, $\varepsilon_{dt},\;E(\varepsilon_{dt}|X_{idt},\pi_{d},\nu_{t})=0$ , denotes an idiosyncratic error that is strictly exogenous. As long as this assumption holds, it is also assumed that ε*_dt_* has a constant variance across time, $\sigma_{e}^{2}$ i.e. no heteroskedasticity, and that the errors are not serially correlated, $E(\varepsilon_{dt}\;\varepsilon_{dt-s})=0$, for all *t≠t-s*. A last assumption that is being made is that there is no multicollinearity in the data.

The model specification as defined in (3) also considers two further potential estimation biases: First, another difficulty in assessing suicide rates with combined aggregate and individual data is that marginal distributions may designate district properties only. In this case, observed effects do not reflect any individual characteristics but rather shifts in suicide rates that depend solely on district assignment. This possibility is avoided though if we assume the district specific suicide rates to be independent of each other because this rules out any unobserved time-constant district heterogeneity. Hence, the baseline probability π*_d_* is defined as a district fixed effect in (3). A second problem has its roots in the time dimension of the data. Suicide rates may change because of events that commonly affect all individuals across all districts at a certain time *t*. These events, i.e. exogenous shocks, represent a source of unobserved district-constant heterogeneity, which causes an additional estimation bias. This bias is accounted for by including a time fixed effect ν*_t_* in (3). This prevents any event at *t* distorting the estimates of suicide risks in the same period.

Generally, the linear specification of *f* (·) greatly simplifies the procedure and allows us to employ the quasi-maximum-likelihood fixed effects estimator for longitudinal spatial error models (SEM) developed by Lee and Yu (61) in order to fit (3).

The models’ residuals were tested for autocorrelation and no autocorrelation was detected by Wooldridge’s autocorrelation test (62), *F*(1,389) = 1.923 (*p* > *F* = 0.166). Moreover, Wald tests were conducted for stepwise model selection based on a basic fixed effect (FE) estimation of (3).

Rather than defining fixed effects, an alternate specification rests on the assumption of π*_d_* as a random effect with *E*(*π_d_* ǀ *X_idt_*,*v_t_*) = 0. Consequently, a random effects estimator (RE) is then applied to (3). In order to compare both estimation strategies a Hausman test (63) was conducted. The results indicate that the fixed effects model is to be preferred over the random effects model, *χ*^2^ = 365.28 (*p* > *χ*^2^ = 0.000). For a further specification test, it was also taken into account that current suicide rates may be influenced by accumulated effects of past levels of SES and SI. We estimated a corrected least squares dummy variable model (LSDVC) (64, 65) including Koyck lags (66) of all independent variables in order to assess long run SES and SI effects on suicide rates. The model yields a mean time of only 1.2 months after which suicide rates are affected by changes in SES and SI levels. Given this high adjustment rate of the model, past SES and SI levels barely contribute to current suicide rates. See the **Supplementary Material** for the results of the additional analyses.

### Limitations

The specification of our model takes account of the functional form of SES and SI effects on the individual level and rules out the estimation of any effects that depend entirely on district assignment. It does not address a further problem associated with ecological data analysis, however. Because of the information on joint distributions lost due to the aggregation of suicides in district rates, effects of district SES and SI compositions cannot be differentiated from individual level SES and SI effects. The estimated coefficients rather represent a blend of individual level and district composition effects (67). Thus, individual level effects are only identified under the untestable assumption that there exist no compositional effects. This assumption is not being made in this study. Since they potentially include individual level effects, significant results are rather regarded as indications for further research. As an additional problem, this also implies that the equations do not identify true treatment effects. Hence, our FE specifications do not reveal causal effects even though they rule out unobserved heterogeneity.

Furthermore, SI should be measured by indicators of social network quality. Such indicators are not available in the data. One-person-households and single marital status were therefore used as proxies for SI based on the assumption that both variables are associated with a comparatively lower social integration. It should be acknowledged, however, that one-person-households and single status are related to each other and, for this reason, might be correlated as well.

## Results

**Table 4** shows the results from the SEM estimation. As can be seen, district suicide rates decrease by −0.39% (*p* < 0.028) with every percentage point increase in incomes, assuming mean sizes of district proportions of singles, individuals living in one-person-households and persons who moved during the year before suicide. Further, an increase in district unemployment proportions by 1% leads to an 1.20% increase (*p* < 0.035) in district suicide rates. Contradicting these results however, it is observed that, given mean district shares of singles, relocated persons and one-person-households again, a 1% increase in CASMIN scores results in an increase of suicide rates by 0.98% (*p* < 0.021).

**Table 4:** SEM longitudinal regressions of district suicide rates on SES and SI.

	Spatial Error Model ln District Suicide Rate	
	%−Change (95% − CI)	p - value
***SES***		
**ISEI**	0.1 (−0.93, 1.14)	(1.000)
**CASMIN**	**0.98 (0.09, 1.88)**	**(0.021)**
**Income**	**−0.39 (−0.76, −0.03)**	**(0.028)**
Minor employment	−0.46 (−1.45, 0.54)	(1.000)
**Unemployed**	**1.20 (0.04, 2.38)**	**(0.035)**
No. Public Transfers	11.98 (−19.66, 56.09)	(1.000)
ALG I/II	0.06 (−0.80, 0.92)	(1.000)
***SI***		
**Moved last Year**	**−2.13 (−3.52, −0.72)**	**(0.000)**
Single	0.46 (−0.42, 1.35)	(0.639)
**One-Person-Household**	**1.65 (0.09, 3.23)**	**(0.033)**
***SES × SI Interactions***		
ISEI (inv.) *×* Single	0.27 (−0.73, 1.28)	(1.000)
CASMIN (inv.) *×* Single	−0.96 (−2.16, 0.26)	(0.297)
**Income (inv.) × Single**	**0.54 (0.01, 1.07)**	**(0.036)**
ISEI (inv.) × One-Person-Household	−1.15(2.34, −0.05)	(0.072)
CASMIN (inv.) × One-Person-Household	−0.72 (−2.43, 1.03)	(1.000)
**Income (inv.) × One-Person-Household**	**−0.98 (−1.79, −0.17)**	**(0.009)**
ISEI (inv.) × Moved last Year	−0.29 (−2.37, 1.84)	(1.000)
**CASMIN (inv.) × Moved last Year**	**3.52 (1.20, 5.91)**	**(0.000)**
Income (inv.) × Moved last Year	0.15 (−0.63, 0.93)	(1.000)
Observations	5070	

The table reports percentage changes of r_dt_ with Bonferroni corrected 95% confidence intervals in square brackets and Bonferroni corrected p-values in parentheses. Control variables are not shown. Results significant at α = 0.05 are printed in bold. “inv.” is abbreviated from inverted.

Source: German Microcensus & Death Record, 1997 to 2010, & 2013 European Standard Population - ed. by the German Federal Statistical Office (DESTATIS), own calculations were performed with STATA 15.0.

Mean values of ISEI, CASMIN and income presumed, a percentage point increase in district population shares of people living in a one-person-household is shown to be associated with an increase in district suicide rates by 1.65% (*p* < 0.033). An increase in persons moving throughout the last year by 1% is found to decrease suicide rates by −2.13% (*p* < 0.000), though.

Regarding interactions of SES and SI, a 1% decrease in the incomes of the respective district’s single persons increases district suicide rates by 0.54% (*p* < 0.036). In addition to this, a decrease in CASMIN scores among individuals with a recent relocation of 1% is associated with an increase in suicide rates by 3.52% (*p* < 0.000). By contrast, a 1% decrease in the incomes of district individuals living in one-person-households results in a −0.98% decrease in suicide rates (*p* < 0.009).

## Discussion

In light of these results, an inverse relation between SES and suicide rates as claimed by Hypothesis I is partly confirmed by the observed unemployment and income effects. This finding is contradicted by the observation that suicide rates increase with increases in CASMIN scores, however. Due to the employed aggregate data, a reasonable explanation for this finding may exist in district compositions: Average CASMIN levels have been steadily increasing over the entire observation period in Germany (68). Given that the specification of our model does not allow to differentiate between effects of individual characteristics and district composition, it can not be excluded by the observed positive effect that the corresponding individuals boast disproportionally low suicide risks. The same reasoning applies to the results in line with Hypothesis I: Given the data, our model specification does not rule out disproportionally low suicide numbers among unemployed individuals and persons with lower household incomes as compared to employees and individuals with higher incomes.

With regard to SI effects on suicide, the coefficient estimates of the district proportion of persons living in one-person-households partly validate an inverse relation between SI and suicide rates as stated by Hypothesis II. This result also confirms Agerbo et al.’s (38) findings. As these observations represent compound individual and district composition effects, it needs to be noted that our results do not eliminate the possibility that the concerning individuals show lower suicide risks if compared to persons who live in more-than-one-person-households. Vice versa, this is also valid for the negative effects on suicides found for district share increases of individuals moving throughout the year preceding suicide, contradicting Hypothesis II: At the district level, high resident turnover rates prevailing in areas with relatively low suicide rates, e.g. rural regions (69, 70), provide a potential explanation for this observation. This does not preclude higher suicide risks among moving as opposed to non-moving individuals, though.

Moreover, the positive effects of income decreases in population shares of single persons on district suicide rates corroborate the *SES* × *SI* interactions proposed by Hypothesis III. Another confirmation is suggested by the estimate that shows an increase in suicide rates to be associated with CASMIN score decreases in persons who moved during the past year. On the contrary, the observed negative effects on suicides of decreases in household incomes of the districts’ persons living in one-person-households clearly contradict the claimed interactions. Once more compositional effects may be a possible reason for the inconsistent observations. Thus, it cannot be ruled out that individuals living in one-person-households who committed suicide show comparatively low income levels. This may hold even true if the means of the respective marginal distributions increase with ascending suicide rates across *N* × *T* = 5,460 district observations. The same argument applies to the findings confirming Hypothesis III: Incomes in single persons and CASMIN scores in relocated individuals might increase in districts with ascending suicide rates considering the aggregate data employed in this study.

In view of the inconsistent results regarding Hypotheses I to III, it is generally recommended that future studies should incorporate individual level data in order to separate individual level from district composition effects and further investigate the robustness of our observations. Nonetheless, the results demonstrating positive SES and SI effects on suicide rates serve as appropriate starting points for the development of suicide prevention strategies: First, our findings suggest interventions to especially target the unemployed, individuals living in one-person-households, persons with low incomes and relocated individuals with lower educational levels. In a second step, specific strategies should be designed in order to cover the needs of the individuals under concern. In order to do so, future work should focus on the causal mechanisms resulting in suicidality. This is best accomplished by applying longitudinal data and modeling life course interactions of adversities and mental health. Depending on the results, respective programs may then include alliances between psychiatric centers and welfare offices lowering the threshold for unemployed and poorly earning individuals to seek counseling and treatment. For socially isolated individuals, effective interventions may encompass approaches aiming at the conditions that favor social isolation within local communities. Respective strategies could also involve the strengthening of communal support systems as well as the improvement of transport services and communication technologies. In addition to this, direct interventions may be established that help individuals in maintaining or developing interpersonal connections (71).

## Author Contributions

A-FN conceptualized the study. A-FN, CR-K, and UH designed the study. A-FN undertook the statistical analyses. A-FN, CR-K, and UH interpreted the data. A-FN drafted the manuscript. CR-K and UH critically revised the draft for important intellectual content. All the authors approved the final version of the manuscript.

## Funding

The authors acknowledge support from the German Research Foundation (DFG) and Leipzig University within the program of Open Access Publishing. Funding for the provision of the Data by the German Federal Statistical Office (DESTATIS) was provided by UH.

## Conflict of Interest

PD Dr. CR-K reports personal fees from Servier, outside the submitted work. Professor Dr. UH reports personal fees from Lundbeck, personal fees from Servier, personal fees from Bayer Pharma, grants from Medice Arzneimittel, and personal fees from Roche, outside the submitted work.

The remaining author declares that the research was conducted in the absence of any commercial or financial relationships that could be construed as a potential conflict of interest.

## Abbreviations

CASMIN, Comparative Analyzes of Social Mobility in Industrial Nations; FE, Fixed Effects (Model); ICD, International Classification of Diseases; ISEI, International Socio-Economic-Index of Occupational Status; LSDVC, Corrected Least Squares Dummy Variable (Model); RE, Random Effects (Model); SEM, Spatial Error Model; SES, Socioeconomic Status; SI, Social Isolation; WHO, World Health Organization.
