# Supplementary material for: Associations of Suicide Rates With Socioeconomic Status and Social Isolation: Findings From Longitudinal Register and Census Data
Source: Front Psychiatry. 2020 Jan 14;10:898. doi: 10.3389/fpsyt.2019.00898 (PMC6971176; doi:10.3389/fpsyt.2019.00898)
Supplement: Supplementary file 1 [file DataSheet_1.pdf]

Supplementary Material: Associations of Suicide Rates with Socioeconomic Status and Social Isolation: Findings from Longitudinal Register and Census Data

1 SUPPLEMENTARY STATISTICAL TESTS & AND MODELS

1.1 Structural Breaks in Suicide Numbers

Following Maag (2008), structural breaks in reported suicide numbers due to ICD revisions can be detected given:

$$\Delta y_{dt} = \phi z_t + v_t + t\gamma_d + \eta_{dt}, \tag{S1}$$

where $\Delta y_{dt} = y_{dt} - y_{dt-1}$. t represents the year, d the district and v_t a time fixed effect. $t\gamma_d$ is a district specific time trend. Further, the dummy z_t indicates the year of the ICD revision. An error is given by $\eta_{dt} \sim N(0, \sigma_\eta^2)$. According to this specification, the coefficients ϕ identify an effect that is induced by an exogenous shock during the year of the ICD revision, the revision itself or both.

	**POLS**<br>Suicides per District<br>Yearly Change	
	**β [95% - CI]**	**p-value**
Year of ICD Revision	-0.38 [-0.87, 0.12]	(0.14)
N	5070	

Notes: 95% confidence intervals are shown in square brackets, *p*-values in parentheses. Results significant at $\alpha = 0.05$ are printed in **bold**.
Source: German Death Record, 1997 to 2010 - ed. by the German federal statistical office (DESTATIS), own calculations were performed with STATA 15.0.

Table S1: Pooled OLS Regression of yearly changes in suicides per district

Table S1 shows the result of a pooled ordinary least squares (POLS) estimation of equation (S1). The year of the ICD revision has no significant effect on changes in reported suicide numbers. Therefore, the codings of ICD versions 9 and 10 can be combined into a single measure of suicides.

1.2 Age- and Gender-Adjustment of District Suicide Rates

The district specific age- and gender-adjusted standardized suicide rate r_{dt} is given by

$$r_{dt} = 100.000 \sum_{g=0}^{1} \sum_{a=1}^{15} \frac{y_{dta,g}}{p_{dta,g}} \frac{p^e_{a,g}}{\sum_{g=0}^{1} \sum_{a=1}^{15} p^e_{a,g}} \tag{S2}$$

d, t, a and g refer to district, time, age and gender-stratum, y is the number of suicides, p and p^e are the raw and European standard populations. Age-strata were generated by grouping all ages < 10 years, 5 age years consecutively > 10 years and all ages > 75 years, resulting in 15 age groups.

1.3 Alternate Model Specifications

1.3.1 Basic Fixed Effects (FE) Model

Table S2 below reports the results of a fixed effects (FE) estimation of equation (3) in the manuscript. Wald tests were conducted in order to asses the joint significance of variable groups. As it is shown, only the SI variables Moved last year, Single and One-Person-Household were not jointly significant. Since these represent the main interest variables, however, it was decided to keep these in the model. The FE model yields effects sizes comparable to the SEM model estimates presented in the main article. In contrast to the SEM specification, effects of singles' incomes on suicide rates are significant at $\alpha = 0.1$ while the general income effect is not significant (p < 0.154).

	FE<br>ln Suicide Rate<br>% - Change [95% - CI]	p-value
SES I		
$F_{Wald}(df\ 389) = 3.31; p > F_{Wald} = 0.02$		
ISEI	0.00 [-0.96, 0.97]	(1.000)
**CASMIN**	**1.24 [0.37, 2.11]**	**(0.000)**
Income	-0.3 [-0.65, 0.05]	(0.154)
SES II		
$F_{Wald}(df\ 389) = 3.38; p > F_{Wald} = 0.01$		
Minor Employment	-0.29 [-1.26, 0.69]	(1.000)
**Unemployed**	**1.59 [0.45, 2.74]**	**(0.000)**
No. Public Transfers	6.35 [-23.72, 48.27]	(1.000)
ALG I/II	-0.11 [-0.96, 0.75]	(1.000)
SI		
$F_{Wald}(df\ 389) = 1.94; p > F_{Wald} = 0.12$		
**Moved last Year**	**-1.39 [-2.75, -0.01]**	**(0.048)**
Single	0.19 [-0.68, 1.07]	(1.000)
**One-Person-Household**	**1.58 [0.04, 3.13]**	**(0.042)**
SES × SI Interactions		
$F_{Wald}(df\ 389) = 2.73; p > F_{Wald} = 0.004$		
ISEI (inv.) × Single	0.34 [-0.59, 1.27]	(1.000)
CASMIN (inv.) × Single	-0.71 [-1.87, 0.46]	(0.846)
Income (inv.) × Single	0.62 [0.1, 1.14]	(0.09)
ISEI (inv.) × One-Person-Household	-0.69 [-1.83, 0.47]	(0.882)
CASMIN (inv.) × One-Person-Household	-0.33 [-1.98, 1.34]	(1.000)
**Income (inv.) × One-Person-Household**	**-1.19 [-1.97, -0.41]**	**(0.000)**
ISEI (inv.) × Moved last Year	-0.9 [-2.94, 1.19]	(1.000)
**CASMIN (inv.) × Moved last Year**	**2.93 [0.73, 5.19]**	**(0.000)**
Income (inv.) × Moved last Year	0.25 [-0.49, 0.99]	(1.000)
Observations	5266	
AIC	1228.3	

Notes: The table reports percentage changes of r_{dt} with Bonferroni corrected 95% confidence intervals in square brackets and Bonferroni corrected p-values in parentheses. Control variables are not shown. Results significant at $\alpha = 0.05$ are printed in **bold**. The results of Wald joint significance tests are indicated above the corresponding variable groups."inv." is abbreviated from inverted.
Source: German Microcensus & Death Record, 1997 to 2010, & 2013 European Standard Population - ed. by the German Federal Statistical Office (DESTATIS), own calculations were performed with STATA 15.0.

Table S2: FE Regressions of *ln* District Suicide Rates on SES and SI

1.3.2 Corrected Least Square Dummy Variables (LSDVC) Model

The observation year provided, fixed effects and spatial error models allow for the estimation of contemporary SES and SI effects, only. In order to assess the impact of past SES and SI levels on current suicide rates, a model including lagged values of X_{idt} needs to be specified. A parsimonious method in this regard is the Koyck model (1954). Accordingly, we firstly assume that all effects of SES and SI on suicide decline by the same constant rate $|\lambda| < 1$ over time. This is reflected in any of the corresponding model's coefficients with lag length s such that $\beta_s = \beta_0\lambda^s$. The Koyck method has the advantage that arbitrary choices of lag length are avoided because it allows for an infinite number of lags: Using Prentice and Sheppard's approach (1995) again and adding lagged values of the independent variables, let

$$ln(r_{dt}) = n_{dt}^{-1} \cdot \sum_{i=1}^{n_{dt}} f(\mu_d + \sum_{s=0}^{\infty} \beta_s X_{idt-s}) + \delta_{dt}, \tag{S3}$$

with a district baseline probability μ_d, an idiosyncratic error δ_{dt} and an identity function $f(\cdot)$. The long run accumulated effect of SES and SI levels on suicide rates $\beta^* = \sum_{s=0}^{\infty} \partial ln(r_{dt})/\partial X_{idt-s} = \sum_{s=0}^{\infty} \beta_s$ can be obtained after applying the following transformation to (S3): Firstly, see that if (S3) is true at time t, it is also true at $t-1$:

$$ln(r_{dt-1}) = n_{dt-1}^{-1} \cdot \sum_{i=1}^{n_{dt-1}} f(\mu_d + \sum_{s=0}^{\infty} \beta_{s-1} X_{idt-s-1}) + \delta_{dt-s-1} \tag{S4}$$

Then multiply (S4) by λ,

$$\lambda ln(r_{dt-1}) = n_{dt-1}^{-1} \cdot \sum_{i=1}^{n_{dt-1}} f(\lambda\mu_d + \sum_{s=0}^{\infty} \lambda\beta_{s-1} X_{idt-s-1}) + \lambda\delta_{dt-s-1}, \tag{S5}$$

and subtract (S5) from (S3). Subsequent rearranging yields:

$$ln(r_{dt}) = \lambda ln(r_{dt-1}) + n_{dt}^{-1} \cdot \sum_{i=1}^{n_{dt}} f(\pi_d + \beta_0 X_{idt}) + \nu_t + \varepsilon_{dt}, \tag{S6}$$

where $\pi_d = \mu_d - \lambda\mu_d$ and $\varepsilon_{dt} = \delta_{dt} - \lambda\delta_{dt-1}$. For the reasons stated in the main article there are also fixed effects π_d and ν_t included. Note now that β^* entails a geometric series that converges to $\beta_0/1-\lambda$. By this means, the long run estimate $\hat{\beta}^*$ can be calculated from the estimated coefficients $\hat{\beta}_0$ and $\hat{\lambda}$. Estimating long run effects, it needs to be considered that $\hat{\beta}^*$ does not provide any information on how long suicide rates take to adjust to changes in X_{idt-s}. In fact, the impact of past values of X_{idt} on suicides decreases with the adjustment rate of the model, that is past levels of SES and SI contribute only little to β^* at high adjustment speeds. In order to assess the average model adjustment time, the mean lag is computed. This is given by $\sum_{s=0}^{\infty} \partial\beta_s/\partial\lambda/\beta^* = \sum_{s=0}^{\infty} s\beta_s/\beta^* = \lambda/1-\lambda$, see also Koyck (1954).

(S6) is being estimated by firstly differencing out the fixed effects. As a result of applying this method it should be pointed out that the error term of the demeaned equation will be correlated with its lagged dependent variable regressor (Nickell, 1981). The resulting bias is shown to decrease with an increasing time dimension of the panel as $N \to \infty$. Concerning the data utilized for this study, Monte Carlo simulations

(Judson and Owen, 1999) suggest that $T = 14$ time periods are to few in order to ignore the bias. Applying the standard FE estimator would therefore turn out inconsistent results. Thus, the Corrected Least Square Dummy Variable (LSDVC) estimator derived by Kiviet (1995) and Bun and Kiviet (2003) is applied to estimate (S6).[1]

As can be seen, the LSDVC model yields effects sizes comparable to the SEM model estimates presented in the main article. The contemporary and long run effects of income on district suicide rates are significant at $\alpha = 0.1$, as opposed to the SEM specification, which turns out an income effect significant at $\alpha = 0.05$.

[1] FE and an OLS regression with district and time dummies are mathematically equivalent.

	LSDVC<br>ln Suicide Rate	
	**% - Change [95% - CI]**	**p-value**
SES		
ISEI	-0.08 [-1.1, 0.96]	(1.000)
**CASMIN**	**1.3 [0.38, 2.23]**	**(0.000)**
Income	-0.34 [-0.71, 0.02]	(0.07)
Minor Employment	-0.31 [-1.27, 0.66]	(1.000)
**Unemployed**	**1.55 [0.38, 2.74]**	**(0.000)**
No. Public Transfers	2.43 [-27.84, 45.38]	(1.000)
ALG I/II	-0.07 [-0.98, 0.86]	(1.000)
SI		
**Moved last Year**	**-1.45 [-2.85, -0.03]**	**(0.042)**
Single	0.35 [-0.54, 1.24]	(1.000)
One-Person-Household	1.49 [-0.16, 3.18]	(0.093)
SES × SI Interactions		
ISEI (inv.) × Single	0.32 [-0.64, 1.29]	(1.000)
CASMIN (inv.) × Single	-0.77 [-2.01, 0.48]	(0.702)
**Income (inv.) × Single**	**0.55 [0.00, 1.09]**	**(0.045)**
ISEI (inv.) × One-Person-Household	-0.83 [-2.04, 0.4]	(0.558)
CASMIN (inv.) × One-Person-Household	-0.43 [-2.18, 1.35]	(1.000)
**Income (inv.) × One-Person-Household**	**-1.042 [-1.88, -0.2]**	**(0.009)**
ISEI (inv.) × Moved last Year	-0.920 [-3.17, 1.39]	(1.000)
**CASMIN (inv.) × Moved last Year**	**3.33 [0.97, 5.75]**	**(0.000)**
Income (inv.) × Moved last Year	0.0550 [-0.74, 0.85]	(1.000)
**ln Suicide Rate - 1st lag**	**0.09 [0.06, 0.12]**	**(0.000)**
Observations	4970	
AIC	1139.2	

Notes: The table reports percentage changes of r_{dt} with Bonferroni corrected 95% confidence intervals in square brackets and Bonferroni corrected p-values in parentheses. Control variables are not shown. Results significant at $\alpha = 0.05$ are printed in **bold**."inv." is abbreviated from inverted.
Source: German Microcensus & Death Record, 1997 to 2010, & 2013 European Standard Population - ed. by the German Federal Statistical Office (DESTATIS), own calculations were performed with STATA 15.0.

Table S3: LSDVC Regressions of *ln* District Suicide Rates on SES and SI

	LSDVC - Long Run Effects ln Suicide Rate	
	**% - Change [95% - CI]**	**p-value**
SES		
$\hat{\beta}^*_{ISEI}$	-0.08 [-1.21, -0.08]	(1.000)
$\boldsymbol{\hat{\beta}^*_{CASMIN}}$	**1.43 [0.42, 2.45]**	**(0.000)**
$\hat{\beta}^*_{Income}$	-0.34 [-0.78, 0.02]	(0.07)
$\hat{\beta}^*_{Minor\,Employment}$	-0.34 [-1.39, 0.73]	(1.000)
$\boldsymbol{\hat{\beta}^*_{Unemployed}}$	**1.7 [0.41, 3.02]**	**(0.000)**
$\hat{\beta}^*_{No.\,Public\,Transfers}$	2.67 [-30.15, 50.92]	(1.000)
$\hat{\beta}^*_{ALGI/II}$	-0.07 [-1.08, 0.94]	(1.000)
SI		
$\boldsymbol{\hat{\beta}^*_{Moved\,last\,Year}}$	**-1.59 [-3.13, -0.04]**	**(0.042)**
$\hat{\beta}^*_{Single}$	0.38 [-0.6, 1.37]	(1.000)
$\hat{\beta}^*_{One-Person-Household}$	1.64 [-0.18, 3.49]	(0.093)
SES × SI Interactions		
$\hat{\beta}^*_{ISEI\,(inv.)\,\times\,Single}$	0.35 [-0.71, 1.42]	(1.000)
$\hat{\beta}^*_{CASMIN\,(inv.)\,\times\,Single}$	-0.85 [-2.21, 0.53]	(0.702)
$\boldsymbol{\hat{\beta}^*_{Income\,(inv.)\,\times\,Single}}$	**0.6 [0.00, 1.2]**	**(0.045)**
$\hat{\beta}^*_{ISEI\,(inv.)\,\times\,One-Person-Household}$	-0.91 [-2.24, 0.45]	(0.558)
$\hat{\beta}^*_{CASMIN\,(inv.)\,\times\,One-Person-Household}$	-0.48 [-2.39, 1.48]	(1.000)
$\boldsymbol{\hat{\beta}^*_{Income\,(inv.)\,\times\,One-Person-Household}}$	**-1.04 [-2.07, -0.22]**	**(0.009)**
$\hat{\beta}^*_{ISEI\,(inv.)\,\times\,Moved\,last\,Year}$	-1.01 [-3.49, 1.53]	(1.000)
$\boldsymbol{\hat{\beta}^*_{CASMIN\,(inv.)\,\times\,Moved\,last\,Year}}$	**3.67 [1.06, 6.36]**	**(0.000)**
$\hat{\beta}^*_{Income\,(inv.)\,\times\,Moved\,last\,Year}$	0.06 [-0.81, 0.93]	(1.000)
Mean Lag Time		
$\boldsymbol{\hat{\lambda}^*}$	**0.1 [0.06, 0.13]**	**(0.000)**

Notes: The table reports percentage changes of r_{dt} with Bonferroni corrected 95% confidence intervals in square brackets, except for $\hat{\lambda}^*$ where it presents the mean lag time in years. Bonferroni corrected p-values are shown in parentheses. Results significant at $\alpha = 0.05$ are printed in **bold**. The CI are based on delta method approximated S.E.. “inv.” is abbreviated from inverted.
Source: German Microcensus & Death Record, Area Population Numbers of Germany, 1997 to 2010, & 2013 European Standard Population - ed. by the German federal statistical office (DESTATIS), own calculations were performed with STATA 15.0.

Table S4: LSDVC Regressions of *ln* District Suicide Rates on SES and SI - Long Run Effects

REFERENCES

Maag T. Economic correlates of suicide rates in OECD countries. Discussion Paper 6, St. Gallen Research Centre for Ageing, Welfare and Labour Market Analysis (SCALA), St. Gallen (2008).

Koyck LM. *Distributed lags and investment analysis* (Amsterdam: North-Holland Publishing Company) (1954).

Prentice R, Sheppard L. Aggregate data studies of disease risk factors. *Biometrika* **82** (1995) 113–125. doi:10.1093/biomet/82.1.113.

Nickell S. Biases in dynamic models with fixed effects. *Econometrica* **49** (1981) 1417–1426. doi:10.2307/1911408.

Judson RA, Owen AL. Estimating dynamic panel data models: A guide for macroeconomists. *Economics Letters* **65** (1999) 9–15. doi:10.1016/s0165-1765(99)00130-5.

Kiviet JF. On bias, inconsistency, and efficiency of various estimators in dynamic panel data models. *Journal of Econometrics* **68** (1995) 53–78. doi:10.1016/0304-4076(94)01643-e.

Bun MJ, Kiviet JF. On the diminishing returns of higher-order terms in asymptotic expansions of bias. *Economics Letters* **79** (2003) 145–152. doi:10.1016/s0165-1765(02)00299-9.
